# The Administration of Chloroform

**Published:** 1880-09

**Authors:** Richard L. Macdonnell


### ARTICLE V.

### *The Administration of Chloroform.*

BY RICHARD L. MACDONNELL, B. A., M. D.

I present the history of an almost fatal case of Chloroform Poisoning, and to venture to make a few remarks on the use of that anæsthetic in Minor Surgery. I do so, not with the idea that any of you will directly gain instruction thereby, but in the hope that some members of this Society, whose experience in the use of anæsthetics will help to throw some light upon a still unsettled question, will instruct the junior members by telling them of the results of their practice, whether they be confirmatory or otherwise of the statements I am about to make.

To begin with, I shall ask you to listen to the history of this case, which I think is one of unusual interest—a case in which, in my own practice, the careful administration of chloroform nearly caused the death of a child.

On the 27th January, 1880, I was to circumcise a boy aged two years and seven months. He was a very healthy child, one of a very healthy family. I have had them all under my care for the last three years. The boy, though he had suffered much pain and inconvenience from the long tight prepuce, was apparently in excellent condition. A few minutes before the operation, I sent to the Medical Hall for one ounce of chloroform, in order that I might be certain that I was using a fresh and reliable material. I made the inhaler myself of a towel. The aperture at the top of the cone was unusually wide. The patient was placed supine at the end of a horizontal operating chair. All his clothes were removed, except flannel shirt. Dr. Sheperd placed twenty minims (by measure) of chloroform on the inhaler. The boy scarcely cried or struggled at all, inhaled freely, and went under the influence of the anæsthetic almost immediately. At the time I thought that he went under too quickly, and I noticed that he was very pale, and that his eyes were upturned. The clamp was applied to the prepuce, and the knife was half way through it, when the boy came to and struggled very violently. Ten minims (by measure) were then added to the towel, and the child again fell asleep, quite suddenly, the pallor and upturning of the eyes being again noticeable. On completing the section of the prepuce and slitting up the mucous membrane, I noticed that the hæmorrhage was very slight. Without further warning, respiration suddenly ceased, and the pulse became imperceptible. There was deadly pallor. Dr. Sheperd immediately inverted the child, and artificial respiration was put into practice. For a time, which of course seemed to me to be hours, but which was, I believe, nearly ten minutes, there was not the slightest response to our efforts. The time passed must have been considerable, for I had time to go out of the room and draw water in the pantry, and afterwards to go out to the back of the house to get snow to throw upon the child's body. I had also to hunt amongst my instruments, to find a forceps to hold up the tongue. At length the application of cold water caused the child to make one little gasp; a second and a third soon followed, and we desisted from our labours with a very hearty sigh of relief. The operation was continued; the child received no more chloroform, but remained asleep for some time time after the last stitch had been put in. For the next forty-eight hours, the child slept almost incessantly.

I do not think that any one can say that the unpleasant event was due to any neglect of necessary precautions. The chloroform was measured, not dropped, and the quantity was noted. The makers were Duncan & Flockhart.

Very few cases of recovery are recorded, after the well-known pallor and cessation of respiration have occurred.

*The American Practitioner* (Feb. 5th, 1875) mentions a case in the practice of Dr. Jordan, Birmingham, Alabama, much resembling this case of mine. The patient, aged eighteen, a girl, had chloroform given to her for tooth extraction. "After four or five inhalations, some spasmodic movements of the face being observed, the napkin was removed, and the patient directed to open her mouth, which she did, when the tooth was extracted without pain." The usual bad symptoms then set in. Inversion was practised, with artificial respiration. In about five minutes there was a faint attempt at breathing, followed, after a long and painful period, by another. As soon as she was put into the horizontal position the breathing again ceased, and the pulse disappeared. Inversion and artificial respiration were again practised, and the patient for the second time revived. Again she was put in a horizontal position, and again she fainted. She finally recovered, after about four hours' careful watching.

I believe it to be the custom of some surgeons to prefer chloroform for minor operations, on account of the facility with which it can be administered, especially, for instance, in tooth drawing. Now, it has been thought that chloroform, in these very operations, is more especially fatal. Any one may see, in the reports of chloroform deaths, the large proportion of deaths during the performance of minor operations. Moreover, some authorities on the subject of anæsthesia think that some particular operations predispose to poisoning. Thus, Dr. Kidd (*Medical Times and Gazette*, Oct. 5, 1861) states that the operations where nearly all the accidents have occurred have been those for removal of toe-nails, dead phalanges, tooth drawing, strabismus, operations on the testis, reduction of dislocations—all more or less connected with tendinous tissue; and to quote Dr. Richardson (*Medical Times and Gazette*, May 28th, 1861,) "I see nothing in operative surgery (except that there seem to have been more deaths from the vapour when it has been used for minor than for major operations) to account for the fatality."

Seeing, then, that great risk attends the administration of chloroform, and that this risk is increased, rather than diminished, by the smallness of the dose and the trivial nature of the intended operation, is it not our duty to put aside anæsthetics as much as possible, and in cases where they must be used, to have recourse to some safer agent?

In fact, I go as far as the editor of the *Medical Times and Gazette*, who says (July 24th, 1875,) "We never see it given to anæsthesia without anxiety, and we are prepared to maintain that its use in any minor operation is quite unjustifiable."

In one of the earliest collections of fatal cases, that of Dr. Crisp, brought before the Medical Society of London in 1852, there were records of twenty deaths. In four of these the chloroform had been given for tooth extraction, and in two others cases for the purpose of procuring sleep.

In Dr. Snow's fifty fatal cases the following operations were about to be performed: extraction of teeth and evulsion of toe-nails were most numerous, then follow fistula, opening of a sinus, ligature of piles, application of potassa fusa to ulcers of the legs, and of nitric acid to venereal sores and warts, (6 cases,) removal of small tumors of the cheek, removal of necrosed bone.

The Committee of the Medical and Chirurgical Society of London, published a list of one hundred and nine fatal cases. And again one cannot help being struck with the number of fatal cases in minor surgery, where probably a very small amount was administered. Thus, there were, out of the hundred and nine operations, five dislocations, nine removals of tumors, three examinations for injuries, twelve operations on the male genito-urinary organs, six applications of escharotics, six plastic operations, twelve tooth extractions, five removals of toe-nails, two cases where it was given for the relief of neuralgia.

The fatal cases of chloroform inhalation collected by Dr. Samson are one hundred and seven in number. In sixty-two of these the anæsthetic was given for minor causes. Thus:

*Minor Causes.*—

| | |
|---|---|
| Relief of Pain—Lead colic, neuralgia, toothache. . . | 4 |
| Examination of injuries and diseases . . . . . . . . . . . | 7 |
| *Minor Operations*—Removal of toe-nails (5;) of testicle (4;) incisions in the perinæum and cauterization (11;) extraction of teeth (8;) fistula (5;) amputation of fingers (4;) of toe; of penis; opening sinus; removing piles; sounding bladder; catheterism; ligature of vessels; phymosis; polypus uteri; circumcision; squint (2;) removal of fœces . . . . . . . . . . . . . . . . . . . . . . . . . . . . | 51 |
| | 62 |

In a table containing the particulars of one hundred and fifty-six deaths from chloroform, collected by Dr. Laurence Turnbull, Aural Surgeon to the Jefferson Medical College Hospital, in Philadelphia, there are fourteen cases of tooth extraction. The eldest of these cases was forty-three. The majority had no lesion to account for the fatal result. Amongst the other causes in this table for which chloroform had been given, there were of ailments, facial neuralgia, asthma, headache, toothache, sleeplessness, uterine trouble; of operations, extractions of teeth as already mentioned; introduction of catheter, extraction of thorn, amputation of finger and toes, hydrocele, removal of dead bone, dressing of fracture tumors of small size, fissures of anus.

Is chloroform a safe anæsthetic for children? I believe I am not in error in saying that the common belief is that children enjoy an immunity from the dangers of chloroform. "Chloroform seems also perfectly safe in children." This statement I quote from Holmes' *Principles and Practice of Surgery.* Snow, writing in 1858, states: "It is worthy of remark that none of the accidents from chloroform which have been recorded have occurred to young children." I believe the prevalence of this opinion is based on the following considerations:

Deaths from chloroform may be divided into two classes, viz: (1) Those cases where an organic lesion is found to account for death; (2) those in which no such lesion is found. Now, children belong to the latter class, it not being a common thing to find heart-walls fattily degenerated in the young. Again, children are less frequently the subject of operation than are adults. I believe that a healthy child and a healthy adult stand an equal chance of poisoning. Chloroform is very frequently given to the young for very trifling reasons—often to allay fright, to satisfy a timid mother, or to gain for the operator the reputation of being a kind-hearted man, unable to witness the sufferings of a child.

Deaths from chloroform in children are more common than is generally supposed.

A case is mentioned in the *Medical Times and Gazette* (March 7th, 1857,) where chloroform given for a nævus operation proved fatal to a child one year old.

Dr. Turnbull, in his *Anæsthetic Manual*, mentions eighteen fatal cases. The ages were one, two, four and a half, six, and the remainder up to twelve. In one patient, aged twelve a tooth was being extracted; another, aged eight, was having a wound dressed, while the operation under which a third, aged eleven, suffered, was for an "injury of the toe." The *Lancet* reports two deaths in 1867, one at University College Hospital, London, and one at Manchester, in children aged respectively eight and nine years. Both operations were for strabismus.

In the case which I have read before you this evening, the patient received altogether the vapour of only thirty minims. This, for a child of two and a half years of age, struck me as being an unusually small amount to produce such alarming symptoms. Indeed, in all the records of fatal cases I have seen, I cannot find any one where the death appears to have been caused directly by an over dose, or where the prolongation of the operation has brought about the fatal result. Dr. Richardson found that in small animals a very heavy vapour of chloroform caused instant death from direct action on the nervous system, and not from absorption into the blood; and after quoting Dr. Snow's table, showing that nine out of fifty cases died within the minute after the commencement of inhalation, and four of these within a few seconds, he goes on to say (*Medical Times and Gazette*, May 28th, 1870:) "The moral of this point is of deep interest, and has at least two meanings. It tells us that chloroform, being a direct irritant, is, in that sense alone, an objectionable narcotic vapour; and it tells us further that it is a bad practice to commence the inhalation too abruptly, or to force on narcotism rudely against time." It seems, therefore that chloroform kills merely by its presence, and that a person on whom, by idiosyncrasy, chloroform has a bad effect, will die as quickly from a small dose as from a large dose."

I have heard people talk of giving a "whiff of chloroform" just as they would talk of giving a patient a "drop of brandy" or a "sip of tea," and yet this whiff has often caused instantaneous death. Dr. Turnbull's 142nd case, a woman, aged twenty-five, with "uterine trouble," had "a whiff" amounting to a few drops. It is true that this woman was found to have had a fatty heart; but on the other hand, she had taken chloroform on a previous occasion without a bad symptom. Fifteen to twenty drops were fatal to the 21st case. Forty drops caused death to the 156th case. This patient, whose age was thirty-eight, was anæsthetized to facilitate the reduction of a dislocation of the shoulder-joint. The heart had been examined before the operation, and was thought to be in a healthy condition, but the autopsy proved that it was fattily degenerated.

Dr. J. D. Gillespie, of Edinburgh, reports (*Lancet*, January 6th, 1864) that he gave a young lady who was timid about the extraction of a tooth, fifty minims of chloroform in two doses. She died, but there were no lesions to account for the fatal result.

I have noticed that in the great majority of fatal cases bad symptoms set in on the administration of the second dose. My patient ceased breathing on the second administration (the ten-minim dose.) This is probably not so much due to the cumulative effects of the agent itself as to the fact that it is usually given in the struggling stage, which is thought to be the most dangerous period.

At Leicester (*Lancet*, September 3rd, 1876,) half a drachm of chloroform caused the death of a man, aged fifty. To quote the comment of the editor of the *Lancet*, " Died from the administration of a dose of chloroform scarcely larger than that which is constantly given by unskilled administrators for the relief of cough and dyspnœa, and the conditions on which death depended were undiscoverable during life."

In Dr. Snow's sixteenth case the patient, aged twenty-four, died after the inhalation of forty minims in two doses. Such are the results which can be brought about by a "whiff of chloroform."

The length of time during which the bad symptoms are present varies in different cases. In this case of mine it seemed very long; but I think the period during which the child did not breathe must have been somewhere between five and ten minutes. Mr. Jonathan Hutchinson (*British Medical Journal*, March 8th, 1873) writes as follows. "In a case of a girl at Reigate, in whom apparent death occurred during excision of the knee, we had to perform artificial respiration for at least *ten* minutes before any signs of reanimation occurred. In another case at the London Hospital, in an amputation of the thigh, the period during which I thought the patient dead was almost as long."

Mr. Lawson Tait (*The Practitioner*, Febuary, 1876) found the treatment of chloroform poisoning by inversion and artificial respiration successful in the case of a girl, aged 18, on whom he was performing Amussat's operation. He thinks it was "fully five minutes before she resumed respiration independently of assistance."—*Canada Med. and Surg. Journal.*

---

### EDITORIAL, ETC.

